# Identification and characteristics of a novel acquired aminoglycoside phosphotransferase, APH(3′)-IVb, from *Riemerella anatipestifer*

**DOI:** 10.1128/aac.01631-25

**Published:** 2026-03-24

**Authors:** Mingkang Zhou, Zhishuang Yang, Mingshu Wang, Renyong Jia, Shun Chen, Mafeng Liu, Xinxin Zhao, Qiao Yang, Ying Wu, Shaqiu Zhang, Juan Huang, Xumin Ou, Di Sun, Bin Tian, Yu He, Zhen Wu, Anchun Cheng, Dekang Zhu

**Affiliations:** 1 Research Center of Avian Diseases, College of Veterinary Medicine, Sichuan Agricultural University 506176 , Chengdu, Sichuan, China; 2 Agricultural Animal Diseases and Veterinary Public Health Key Laboratory of Sichuan Province, Chengdu, Sichuan, China; 3 Key Laboratory of Agricultural Bioinformatics, Ministry of Education, Ministry of Education of the People’s Republic of China, Chengdu, Sichuan, China; 4 International Joint Research Center for Animal Disease Prevention and Control of Sichuan Province, Chengdu, Sichuan, China; 5 Department of Animal Science and Technology, Chongqing Three Gorges Vocational College 590168 , Chongqing, China; 6 Engineering Research Center of Southwest Animal Disease Prevention and Control Technology, Ministry of Education of the People’s Republic of China, Chengdu, Sichuan, China; 7 Veterinary Department, College of Animal Science, State Key Laboratory of Green Pesticide, Institute of Veterinary Immunology and Green Drugs 71206, Guiyang, Guizhou, China; Entasis, Big Bay, Michigan, USA

**Keywords:** *Riemerella anatipestifer*, APH(3′)-IVb, antimicrobial resistance, aminoglycoside phosphotransferase

## Abstract

A novel acquired aminoglycoside resistance gene, *aph(3*′*)-IVb*, was identified via whole-genome sequencing of a multidrug-resistant *Riemerella anatipestifer* isolate from a duck. The gene encodes a 262-amino-acid phosphotransferase, APH(3′)-IVb, sharing only 39.9% amino acid identity with its closest known homolog, APH(3′)-IVa. Heterologous expression of *aph(3*′*)-IVb* in *Escherichia coli* and a susceptible *R. anatipestifer* strain conferred resistance to neomycin, paromomycin, and ribostamycin, a phenotype validated by gene deletion and complementation experiments. Kinetic analysis of the purified APH(3′)-IVb enzyme confirmed phosphotransferase activity against these three aminoglycosides, with catalytic efficiencies (*k*_cat_/*K_m_*) ranging from 10⁴ to 10⁵ M⁻¹·s⁻¹. Furthermore, site-directed mutagenesis identified key residues critical for enzymatic function. While the prevalence of *aph(3*′*)-IVb* in *R. anatipestifer* isolates was low (1.6%), analysis of public databases identified 93 *aph(3*′*)-IVb*-positive sequences, of which 36.6% originated from human pathogens. Genetic environment analysis revealed that *aph(3*′*)-IVb* resides within a genomic resistance island flanked by mobile genetic elements, suggesting its horizontal acquisition. The emergence of this novel enzyme, coupled with its association with mobile elements and distribution among human pathogens, underscores a potential pathway for resistance dissemination across veterinary and clinical environments, posing a significant public health concern.

## INTRODUCTION

Aminoglycoside antibiotics remain cornerstones of antimicrobial therapy, widely utilized for treating severe bacterial infections due to their broad-spectrum potency (1, 2). The primary determinant of clinical resistance to these agents is the enzymatic inactivation of the drug, mediated by aminoglycoside-modifying enzymes (AMEs) (3, 4). AMEs are categorized into three major families—N-acetyltransferases, O-nucleotidyltransferases, and aminoglycoside O-phosphotransferases (APHs)—based on the specific chemical modification (acetylation, adenylation, or phosphorylation, respectively) they catalyze (2, 5, 6). Nomenclature within these families is defined by the site of modification (e.g., 3′, 6′, or 2″), followed by a Roman numeral indicating the specific resistance profile and a lowercase letter denoting the unique protein variant (5, 6). Among the nearly 40 identified APH enzymes, the APH(3′) family is the most diverse, comprising seven subclasses (I–VII) with distinct substrate specificities (7, 8). Specifically, the APH(3′)-IV subclass is characterized by its resistance profile against neomycin (NEO), paromomycin (PAR), and ribostamycin (RIB) (5). Historically, *aph(3*′*)-IVa* has represented the sole documented gene within this subclass (9). Initially isolated from *Niallia circulans*, this gene has been extensively characterized regarding its sequence, heterologous expression, and regulatory elements.

*Riemerella anatipestifer*, a gram-negative bacterium of the family *Weeksellaceae*, is the etiological agent of infectious serositis (10). This highly contagious pathogen affects a broad range of avian hosts—including ducks, geese, and turkeys—and is responsible for significant outbreaks worldwide. Clinical manifestations range from acute septicemia to chronic polyserositis (characterized by fibrinous pericarditis, perihepatitis, and salpingitis) and meningitis. These infections result in high mortality rates and growth retardation, imposing a substantial economic burden on the global poultry industry (11). While antimicrobials such as aminoglycosides, florfenicol, and macrolide-lincosamide-streptogramin B agents are routinely employed for disease control (12), their extensive use has driven the selection and acquisition of resistance determinants. Consequently, the continuous evolution of the *R. anatipestifer* resistome has led to the emergence of multidrug-resistant (MDR) isolates (13). High-level resistance to diverse antibiotic classes, including quinolones, aminoglycosides, and tetracyclines, is now prevalent among clinical isolates (14). Furthermore, intrinsic resistance mechanisms, particularly the ubiquitous resistance-nodulation-division (RND) and ATP-binding cassette (ABC) efflux pumps, provide a basal level of protection against aminoglycosides and organic solvents in this species (12, 14).

In this study, we report the identification and functional characterization of a novel APH(3′)-IV variant, designated APH(3′)-IVb. The *aph(3*′*)-IVb* gene resides within a novel acquired genomic resistance island in a multidrug-resistant *R. anatipestifer* clinical isolate.

## RESULTS AND DISCUSSION

### Antibiotic susceptibility of *R. anatipestifer* isolate RCAD1101

*R. anatipestifer* RCAD1101 was isolated from the respiratory tract of a diseased duck on a farm in Yunnan Province, China. The minimum inhibitory concentrations (MICs) of aminoglycosides for RCAD1101, the deletion mutant RCAD1101Δ*aph(3*′*)-IVb*, the complementation strain RCAD1101Δ*aph(3*′*)-IVb*(pLMF03-*aph(3*′*)-IVb*), and recombinant strains are summarized in Table 1. Notably, the deletion mutant RCAD1101Δ*aph(3*′*)-IVb* still exhibited high background resistance to neomycin (128 mg/L) and, unexpectedly, an even higher level to ribostamycin (512 mg/L). Although such high-level resistance is often clinically associated with 16S ribosomal RNA (rRNA) methylases, our genomic analysis (Table S3) confirmed the absence of such genes. Furthermore, while the aminoglycoside nucleotidyltransferase gene *aadS* was identified in the genome, it specifically confers resistance to streptomycin and does not contribute to the resistance against 4,5-disubstituted aminoglycosides (e.g., ribostamycin) observed here (15). Thus, this residual resistance is attributed to the intrinsic multidrug-resistance background of *R. anatipestifer*, driven by functionally characterized efflux systems, such as the RND-type RaeABCR and RaeE-RaeF-RopN complexes, and the ABC-type RanARanB system (12, 14, 16), which appear to confer high baseline protection in both the mutant and the standard strain ATCC 11845. Consequently, *aph(3*′*)-IVb* functions as a “resistance amplifier,” acting on top of this intrinsic efflux activity to boost resistance to clinically high levels (≥1,024 mg/L). The MIC values for an additional 19 antibiotics tested against RCAD1101, with interpretive categories, are presented in Table S2. Based on these MIC data, RCAD1101 was resistant to the majority of antibiotics, confirming its classification as an MDR strain.

**TABLE 1:** Aminoglycosides MICs for *R. anatipestifer* and recombinant *E. coli* strains^*a*^

Strains	MIC (mg/L)												
	NEO	PAR	RIB	AMK	SPE	MCR	KAN	NET	SIS	TOB	APR	STR	GEN
*R. anatipestifer* RCAD1101	1,024	1,024	4,096	128	128	32	512	128	256	512	512	64	128
RCAD1101△*aph(3*′*)-IVb*	128	256	512	128	128	32	512	128	256	256	512	64	128
RCAD1101△*aph(3*′*)-IVb*(pLMF03*-aph(3*′*)-IVb*)	1,024	1,024	4,096	128	128	32	512	128	256	256	512	64	128
*R. anatipestifer* ATCC 11845	128	256	512	64	64	64	512	128	256	512	512	256	32
ATCC 11845/pLMF03	128	256	512	64	128	64	512	128	128	512	512	256	64
ATCC 11845/pLMF03- *aph(3*′*)-IVb*	1,024	1,024	4,096	64	128	64	512	128	128	512	512	256	64
*E. coli* BL21	32	32	64	32	64	32	32	8	8	16	32	32	16
BL21/pET32a	32	32	64	32	128	16	32	8	8	8	32	32	16
BL21/pET32a-*aph(3*′*)-IVb*	256	512	＞4,096	32	128	16	32	8	8	8	16	16	16

^*a*^ NEO, neomycin; PAR, paromomycin; RIB, ribostamycin; AMK, amikacin; SPE, spectinomycin; MCR, micronomicin; KAN, kanamycin; NET, netilmicin; SIS, sisomicin; TOB, tobramycin; APR, apramycin; STR, streptomycin; GEN, gentamicin.

### Genome screening and identification of the novel resistance gene *aph(3′)-IVb*

To elucidate the genetic basis of the MDR phenotype of RCAD1101, we sequenced its complete genome (GenBank accession no. CM136845.1) and analyzed it using AMRFinderPlus (version 3.10.24) (17) to identify potential resistance determinants. The screening revealed a total of 38 known and putative resistance genes (Table S3). Among these, a putative novel resistance determinant showing limited homology to characterized aminoglycoside phosphotransferases was identified and designated as *aph(3′)-IVb*. Consequently, subsequent experiments focused on the functional characterization of this novel gene.

### Functional characterization of *aph(3′)-IVb* and its contribution to aminoglycoside resistance

The *aph(3*′*)-IV-like* gene consists of 789 bp and encodes a 262-amino acid protein. It shares the highest amino acid sequence identity (39.9%) with the chromosomally encoded aminoglycoside phosphotransferase APH(3′)-IVa from *Niallia circulans* (9). When expressed in *Escherichia coli* BL21 and *R. anatipestifer* ATCC 11845 (Table 1), the APH(3′)-IV-like protein (designated APH(3′)-IVb) conferred resistance to neomycin, paromomycin, and ribostamycin, but not to tobramycin, sisomicin, netilmicin, spectinomycin, apramycin, gentamicin, amikacin, micronomicin, streptomycin, or kanamycin. Additionally, deletion of the gene resulted in increased susceptibility to these three antibiotics, whereas complementation restored resistance to neomycin, paromomycin, and ribostamycin, confirming the predicted function of *aph(3*′*)- IVb*.

**Fig 1:**
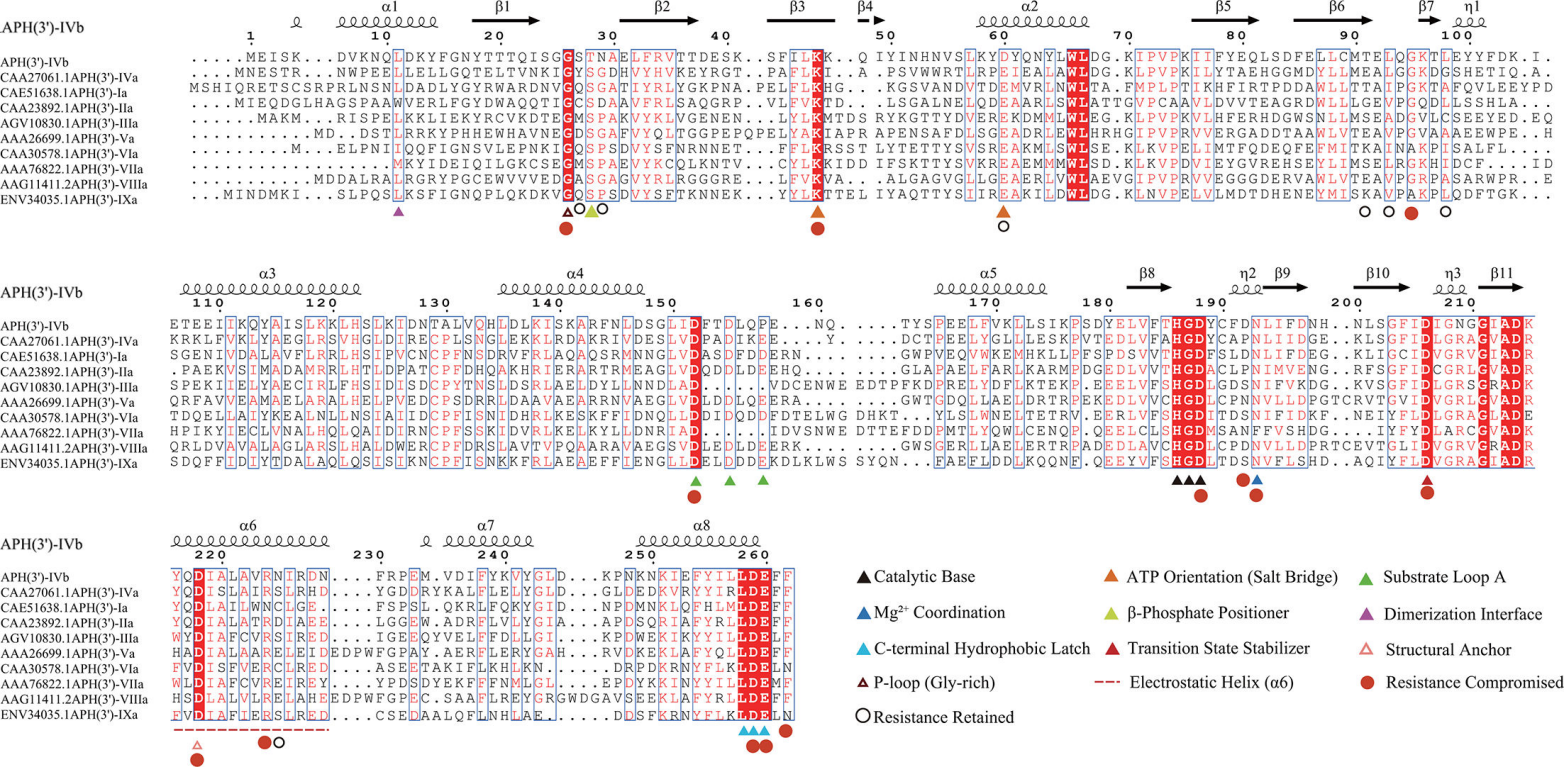
Multiple sequence alignment and structural annotation of APH(3′)-IVb. The red regions indicate fully conserved sites. Colored triangles represent specific functional motifs corresponding to characterized residues in APH(3′) homologs: black triangles indicate the catalytic base essential for phosphate transfer; red triangles mark the catalytic core residue stabilizing the transition state; orange triangles denote the conserved Lys-Glu salt bridge required for ATP orientation; blue triangles identify the invariant Asn residue responsible for coordinating magnesium ions (Mg2^+^); lime triangles indicate the residue positioning the beta-phosphate of ATP; dark red triangles mark the Gly-rich P-loop involved in nucleotide binding; purple triangles show hydrophobic residues forming the dimerization interface; green triangles highlight substrate Loop A defining substrate specificity; cyan triangles mark the C-terminal hydrophobic latch essential for pocket integrity; and pink triangles indicate the structural anchor Asp218 maintaining helix integrity. The red dashed line underscores the electrostatic substrate-binding helix (α6) responsible for recruiting polycationic antibiotics. Circles represent the mutation sites in this experiment, with red filled circles marking the sites where the drug resistance phenotype was compromised after mutation, and open circles indicating the sites where the drug resistance phenotype remained unchanged. The numbers correspond to the positions of amino acid residues in APH(3′)-IVb.

To definitively classify this enzyme given its relatively low sequence identity (39.9%) to APH(3′)-IVa (9), we integrated phylogenetic, sequence alignment, and structural analyses. The phylogenetic tree indicated that this protein clusters within the APH(3′)-IV-type clade (Fig. S1). Consistent with this clustering, multiple sequence alignment revealed that despite the low overall sequence identity, the critical functional motifs characteristic of the APH family are strictly conserved (Fig. 1). These include the critical residues responsible for ATP binding, the catalytic center, and substrate recognition loops, as well as other conserved functional elements, which have been functionally defined in structurally characterized homologs such as APH(3′)-IIIa and APH(3′)-IIa (18–20).

To further validate this classification, we examined the tertiary structure. Structural superposition analysis provided robust complementary evidence. As shown in Fig. S2, the predicted 3D structure of this novel enzyme aligns well with APH(3′)-IVa, yielding a root mean square deviation of only 1.141 Å (21, 22). The observed structural congruity confirms that despite sequence divergence, the enzyme adopts the canonical catalytic fold of the APH(3′)-IV subfamily. Therefore, strictly adhering to the standard classification criteria for aminoglycoside modifying enzymes, which prioritize regioselectivity and substrate profile (5), we designated this gene as *aph(3*′*)-IVb*. This classification is supported by (i) its specific resistance to 4,5-disubstituted aminoglycosides (phenotype), (ii) its highly conserved 3D structure (mechanism), and (iii) its monophyletic clustering (phylogeny). The nomenclature has been submitted to the National Center for Biotechnology Information (NCBI) GenBank database (accession no. PX763553.1).

It is noteworthy that while the prototype APH(3′)-IVa has been reported to confer resistance to a broad spectrum including kanamycin (23), our identified APH(3′)-IVb exhibited a distinct profile limited to neomycin, paromomycin, and ribostamycin. This discrepancy can be reconciled by the evolutionary origins and established classification of this subclass. Foundational studies by Herbert et al. (9, 24) established that the prototype gene from *Bacillus circulans* functions primarily as a self-defense mechanism against butirosin, a member of the neomycin (4,5-disubstituted) family. Furthermore, nomenclature consensus established by Ramirez and Tolmasky (5) explicitly defines the APH(3′)-IV subclass by its resistance to neomycin and ribostamycin, excluding the kanamycin from its core defining spectrum. The high intrinsic resistance to kanamycin (MIC = 512 mg/L) in *R. anatipestifer* further obscures any potential contribution of APH(3′)-IVb to this specific drug. Therefore, the “IVb” designation accurately reflects an enzyme that adheres to the ancestral substrate specificity of the APH(3′)-IV lineage while lacking the expanded phenotype reported in some clinical isolates.

### Kinetic parameters of APH(3′)-IVb

The phosphotransferase activity and kinetic parameters of purified APH(3′)-IVb were determined using purified enzyme and various aminoglycoside antibiotics substrates. Consistent with MIC data (Table 1), the enzyme efficiently phosphorylated neomycin, paromomycin, and ribostamycin, but no activity was detected against amikacin, gentamicin, streptomycin, kanamycin, or sisomicin (Table 2). Direct kinetic comparison with the homolog APH(3′)-IVa was not feasible, as its activity was previously assessed solely via semi-quantitative phosphocellulose paper binding assays without determining substrate affinity (*K*_m_) or catalytic efficiency (*k*_cat_).

**TABLE 2:** Kinetic parameters of APH(3′)-IVb^*a*^ with different aminoglycoside substrates^*c*^

Substrate	*K_m_* (μM)^*b*^	*k*_cat_ (s^−1^)^*b*^	*k*_cat_/*K*_*m*_ (M^−1^/s^−1^)
NEO	22.13 ± 2.08	4.85 ± 0.01	(2.19 ± 0.20) × 10⁵
PAR	60.10 ± 3.12	10.12 ± 0.15	(1.69 ± 0.07) × 10^5^
RIB	54.20 ± 4.93	4.96 ± 0.12	(9.15 ± 0.85) × 10^4^

^*a*^ The protein was initially modified by a His_6_ tag, which was removed after purification.

^*b*^ *k*_cat_ and *K_m_* values represent the mean ± SD of three independent experiments.

^*c*^ NEO, neomycin; PAR, paromomycin; RIB, ribostamycin.

To contextualize these findings, we compared the kinetic profile of APH(3′)-IVb with that of APH(3′)-Ie, a homolog possessing a distinct resistance profile (25). A distinct kinetic trend emerged: APH(3′)-IVb consistently displayed higher turnover numbers (*k*_cat_) but lower substrate affinities (higher *K_m_*) than APH(3′)-Ie across shared substrates. Specifically, for neomycin, APH(3′)-IVb exhibited a 2.96-fold higher *k*_cat_. Although its *K_m_* (22.13 μM) was 1.83-fold higher than that of APH(3′)-Ie (12.07 μM), the overall catalytic efficiency (*k*_cat_/*K*_*m*_) of APH(3′)-IVb remained 2.25-fold superior (2.19 × 10⁵ vs 1.36 × 10⁵ M⁻¹·s⁻¹). Similarly, for paromomycin, APH(3′)-IVb demonstrated an 11.77-fold higher *k*_cat_ (10.12 vs 0.86 s⁻¹) and a 1.48-fold higher catalytic efficiency (1.69 × 10⁵ vs 1.14 × 10⁵ M⁻¹·s⁻¹), despite a 7.76-fold increase in *K_m_*. In contrast, for ribostamycin, while APH(3′)-IVb showed a 3.79-fold higher *k*_cat_ (4.96 s⁻¹), its affinity was markedly lower (*K_m_* = 54.2 µM) compared to APH(3′)-Ie (*K_m_* = 4.22 µM). Consequently, APH(3′)-Ie exhibited a 3.52-fold higher catalytic efficiency for this substrate (3.23 × 10⁵ vs 9.15 × 10⁴ M⁻¹·s⁻¹).

### Site-directed mutagenesis analysis of APH(3′)-IVb

To identify residues critical for APH(3′)-IVb catalysis, we selected 20 positions for alanine scanning mutagenesis. Site selection was guided by multiple sequence alignment with other APH(3′) enzymes, molecular docking models of the APH(3′)-IVb-aminoglycoside complex (Fig. S3), and prior literature (26). Following established protocols, each target residue was individually substituted with alanine. The mutagenesis results (Table 3) revealed three distinct phenotypic classes regarding APH(3′)-IVb-mediated resistance to ribostamycin, paromomycin, and neomycin in *E. coli*.

**TABLE 3:** Aminoglycoside MICs for *E. coli* expressing APH(3′)-IVb site-directed mutants^*a*^

Strains	MIC (mg/L)		
	NEO	PAR	RIB
*E. coli* BL21	32	32	64
BL21/pET32a	32	32	64
BL21/pet32a-*aph(3*′*)-IVb*	256	512	>4,096
BL21/pet32a-*aph(3*′*)-IVb*(E206A)	32	32	64
BL21/pet32a-*aph(3*′*)-IVb*(K46A)	32	32	64
BL21/pet32a-*aph(3*′*)-IVb*(D188A)	32	32	64
BL21/pet32a-*aph(3*′*)-IVb*(N193A)	32	32	256
BL21/pet32a-*aph(3*′*)-IVb*(E260A)	32	32	64
BL21/pet32a-*aph(3*′*)-IVb*(D259A)	32	32	64
BL21/pet32a-*aph(3*′*)-IVb*(G95A)	32	32	512
BL21/pet32a-*aph(3*′*)-IVb*(D155A)	64	256	2,048
BL21/pet32a-*aph(3*′*)-IVb*(△F262)	32	128	512
BL21/pet32a-*aph(3*′*)-IVb*(R224A)	32	32	128
BL21/pet32a-*aph(3*′*)-IVb*(G26A)	64	64	512
BL21/pet32a-*aph(3*′*)-IVb*(D192A)	32	32	512
BL21/pet32a-*aph(3*′*)-IVb*(D218A)	32	32	64
BL21/pet32a-*aph(3*′*)-IVb*(D60A)	256	512	>4,096
BL21/pet32a-*aph(3*′*)-IVb*(T97A)	256	512	>4,096
BL21/pet32a-*aph(3*′*)-IVb*(L93A)	256	512	>4,096
BL21/pet32a-*aph(3*′*)-IVb*(T91A)	256	512	>4,096
BL21/pet32a-*aph(3*′*)-IVb*(S27A)	256	512	>4,096
BL21/pet32a-*aph(3*′*)-IVb*(N29A)	256	512	>4,096
BL21/pet32a-*aph(3*′*)-IVb*(N225A)	256	512	>4,096

^*a*^ NEO, neomycin; PAR, paromomycin; RIB, ribostamycin.

Group I comprised mutations that completely abolished resistance. Substitutions at positions K46, D188, D192, E206, D218, D259, and E260 yielded MICs indistinguishable from the negative control. These results indicate that these residues are essential for the enzyme’s function, as their mutation resulted in a complete loss of resistance to all three tested aminoglycosides.

Group II comprised mutations that partially impaired resistance. Specifically, mutations N193A, G95A, and R224A abolished resistance to NEO and PAR, while the MIC for RIB decreased to ≤128 mg/L (a 32-fold reduction compared to the wild-type strain). The deletion of F262 (ΔF262) eliminated resistance to NEO, reduced the PAR MIC to 128 mg/L (25% of wild-type levels), and decreased the RIB MIC to ≤512 mg/L. Furthermore, G26A and D155A mutations caused a general reduction in resistance to all three antibiotics, with MICs dropping to 50%–12.5% of wild-type levels.

Group III comprised mutations that did not alter resistance. Substitutions at S27, N29, D60, T91, L93, T97, N225, and F262A produced MICs indistinguishable from the wild type, suggesting that these specific side chains are not strictly required for APH(3′)-IVb-mediated resistance under the tested conditions.

Structural comparisons with APH(3′)-IIIa further elucidated these findings. Previous studies have established that APH(3′)-IIIa shares structural homology with eukaryotic protein kinases (18, 27). Sequence alignment identifies five highly conserved residues in APH(3′)-IIIa—Lys44, Glu60, Asp190, Asn195, and Asp208—which correspond to Lys46, Asp60, Asp188, Asn193, and Asp206 in APH(3′)-IVb (Fig. 1), respectively. In APH(3′)-IIIa, Lys44 is critical for catalysis by forming ionic interactions with ATP phosphates (27); consistent with this, the K46A mutation in APH(3′)-IVb completely abolished resistance. Conversely, Glu60 in APH(3′)-IIIa stabilizes the ATP-binding pocket via a salt bridge but is not catalytic (18); similarly, the D60A mutation in APH(3′)-IVb did not significantly compromise resistance. The catalytic core residues (Asp190, Asn195, and Asp208 in IIIa) are essential for substrate positioning and transition state stabilization (18); our data confirmed this conservation in APH(3′)-IVb, as mutations at the corresponding sites (Asp188, Asn193, and Asp206) resulted in abolished or severely impaired resistance.

Regarding the C-terminal loop region, Kaul et al. (19) demonstrated its importance for drug binding in APH(3′)-IIIa (residues 260–264). In APH(3′)-IVb, the homologous residues are Asp259, Glu260, and Phe262. The D259A and E260A mutations in APH(3′)-IVb completely abolished resistance, mirroring the role of their homologs in APH(3′)-IIIa in engaging amino groups of the substrate. For Phe262, our results showed that while the deletion (ΔF262) significantly impaired resistance, the single substitution F262A had no effect. This aligns with findings for Phe264 in APH(3′)-IIIa, where the residue’s presence is crucial for maintaining pocket integrity through steric effects rather than specific side-chain chemistry (19).

Finally, among the remaining residues, G26, G95, D155, D218, and R224 are highly conserved across APH(3′) enzymes, and our results confirmed their functional importance. Notably, although Asp192 (D192) is not conserved in the primary sequence alignment, molecular docking identified it as a key site for ADP/ATP binding. The D192A mutation abolished resistance to NEO and PAR and reduced RIB resistance by ≥8-fold. Thus, D192 likely represents a unique and critical determinant of APH(3′)-IVb-mediated aminoglycoside resistance.

### Prevalence of *aph(3′)-IVb* in *R. anatipestifer* field isolates and its distribution across ecological niches

To investigate the prevalence of *aph(3*′*)-IVb* in clinical settings, we screened 741 *R. anatipestifer* field isolates collected from major poultry-farming provinces in China (Sichuan, Shandong, Jiangxi, Yunnan, and Guangdong). PCR analysis identified 14 positive isolates, corresponding to a detection rate of 1.9% (Table S4). The earliest *aph(3*′*)-IVb*-positive strain in our collection dates back to 5 July 2010. Despite the relatively low overall prevalence, the identification of positive isolates across diverse geographical regions over a 15-year span indicates that the gene demonstrates a capacity for persistence and transmission within the *R. anatipestifer* population.

To broaden our understanding of its ecological distribution, we retrieved 93 sequences sharing > 98% protein sequence identity with APH(3′)-IVb from public databases (Table S5). Note that one sequence was curated as two segments due to a premature stop codon. These sequences originated from at least 23 distinct species, spanning a collection period from 1988 to 2025. The geographical distribution of these strains is illustrated in Fig. S4, with the majority (85/93) originating from China, followed by sporadic isolates from the United States, Bangladesh, Denmark, India, Israel, Peru, and Vietnam. The earliest recorded *aph(3*′*)-IVb*-positive strain was *Parabacteroides goldsteinii* BFG-241 (accession no. NZ_CP081906.1), isolated from a human patient with a Bacteroides infection in the United States (28).

Ecological niche analysis classified these 93 strains into 3 categories: environmental sources (*n* = 5), pathogenic bacteria explicitly associated with disease (*n* = 43), and host-associated commensals (*n* = 45). Among the 43 pathogenic isolates, sources included humans (*n* = 34), ducks (*n* = 7), *Gallus gallus* (*n* = 1), and *Sus scrofa domesticus* (*n* = 1). Notably, the 34 human-derived pathogenic strains were primarily isolated from sputum, blood, and urine samples, suggesting that the gene is frequently detected in human clinical isolates.

Further analysis revealed a strong link between *aph(3*′*)-IVb* and the tigecycline resistance gene *tet*(X). Thirty of the 34 human pathogenic strains were derived from a single study by Zhang et al. (29) and belonged to the phylum Bacteroidota (specifically *Chryseobacterium bernardetii*, *Chryseobacterium indologenes*, *Elizabethkingia meningoseptica*, and *Sphingobacterium mizutaii*); all were confirmed *tet*(X)-positive. Similarly, 28 additional strains (27 host-associated and 1 environmental) identified in other studies (30–32) were also *tet*(X)-positive. This high rate of co-occurrence suggests that the dissemination of *aph(3*′*)-IVb* may be driven by co-selection pressure exerted by the combined clinical use of tetracyclines and aminoglycosides.

Regarding *R. anatipestifer* specifically, public databases contained two positive strains (R-4 and R-17) of duck origin. Combining these with our field surveillance data, the overall prevalence of *aph(3*′*)-IVb* in the *R. anatipestifer* population is estimated at 1.6% (16/975).

### Genetic environment analysis and natural transformation of aph(3′)-IVb

**Fig 2:**
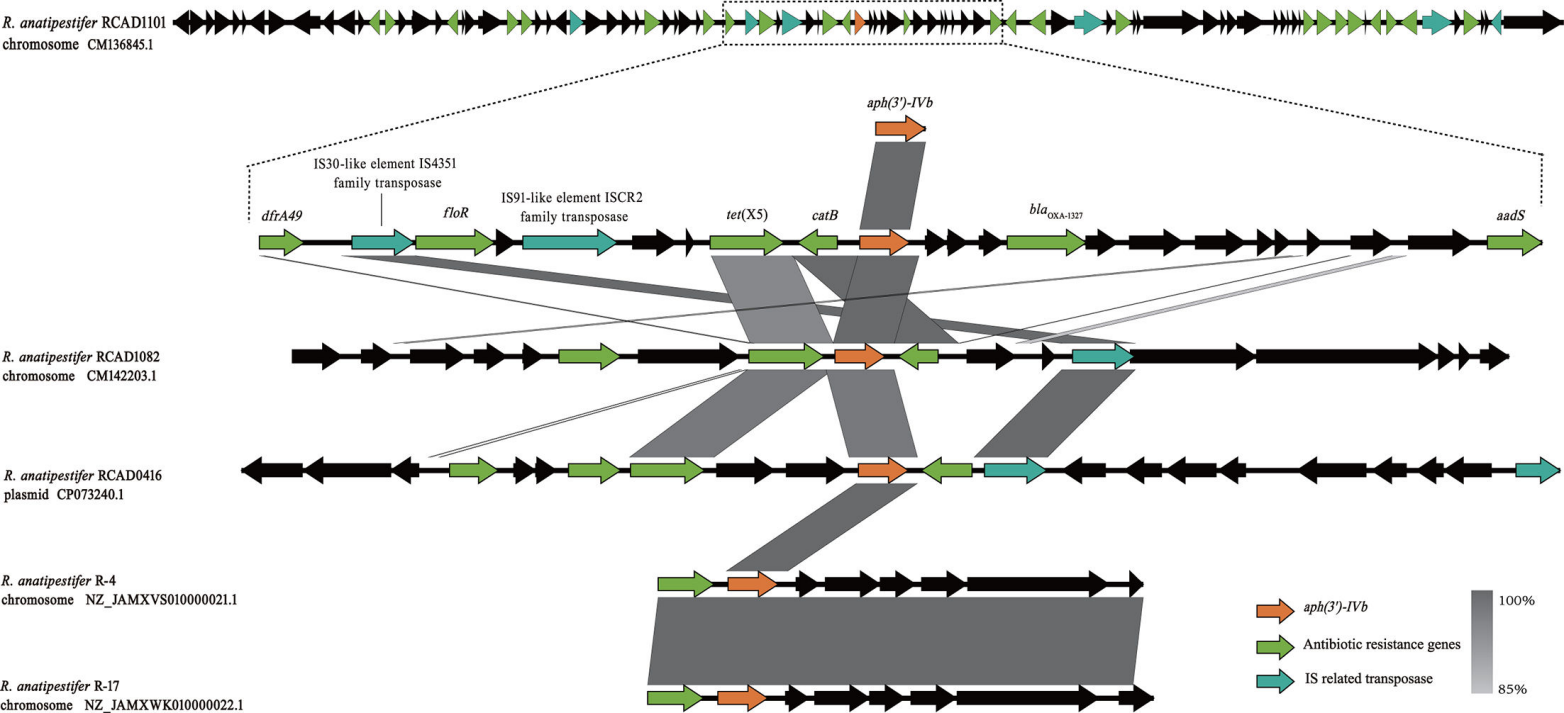
Genetic context of the *aph(3′)-IVb* gene in *R. anatipestifer*. Schematic representation of the genetic environment of *aph(3′)-IVb* and comparison of the *aph(3′)-IVb*-carrying regions in genomes of *R. anatipestifer* strains. Open reading frames are shown as arrows drawn to scale to indicate the direction of transcription. The *aph(3′)-IVb* gene is colored in orange, the known resistance genes are colored in green, the mobile elements are colored in sky blue, and the other genes are colored in black. *dfrA49,* dihydrofolate reductase A49*; floR*, florfenicol resistance gene R; *te*t(X5)*,* tetracycline resistance gene X5; *catB*, chloramphenicol acetyltransferase B; *bla*_OXA-1327_*,* β-lactamase OXA-1327; *aadS* aminoglycoside 6′-N-acetyltransferase S; *lnu*(I), lincosamide nucleotidyltransferase I; *bla*_OXA-347_*,* β-lactamase OXA-347; *tet*(X4)*,* tetracycline resistance gene X4; *erm(F)*, erythromycin resistance methylase F.

To elucidate the genetic context of *aph(3*′*)-IVb* in *R. anatipestifer*, we performed a fine-scale structural analysis of the 100 kb sequences flanking the gene in clinical strains (Fig. 2). This region contains a high density of antimicrobial resistance genes interspersed with mobile genetic elements (MGEs). To assess the novelty of this genomic island, we performed a BLASTn search (Megablast algorithm) against the NCBI nucleotide database. The analysis revealed that no existing entry in the database exhibits complete homology to this region, with the highest query coverage being only 54% (identified in *R. anatipestifer* strain RAf490, GenBank accession no. CP175957.1). Consequently, we identified this region as a novel genomic resistance island (GRI) characterized by a unique mosaic structure.

Specifically, in the 20 kb flanking regions adjacent to *aph(3*′*)-IVb* in strain RCAD1101, a multidrug-resistance cluster was identified, comprising *dfrA49* (sulfonamide resistance), *floR* (phenicol resistance), *tet*(X5) (tetracycline resistance), *aadS* (streptomycin resistance), *catB* (chloramphenicol resistance), and *bla*_OXA-1327_ (β-lactam resistance). Furthermore, two MGEs were characterized adjacent to the gene: an IS30-like insertion sequence (designated IS4351) and an IS91-like insertion sequence (designated ISCR2) both located upstream. Comparative analysis across strains RCAD1101, RCAD1082, and the plasmid-borne *aph(3*′*)-IVb* in RCAD0416 consistently detected IS4351 in the upstream and downstream regions (Fig. 2).

**Fig 3:**
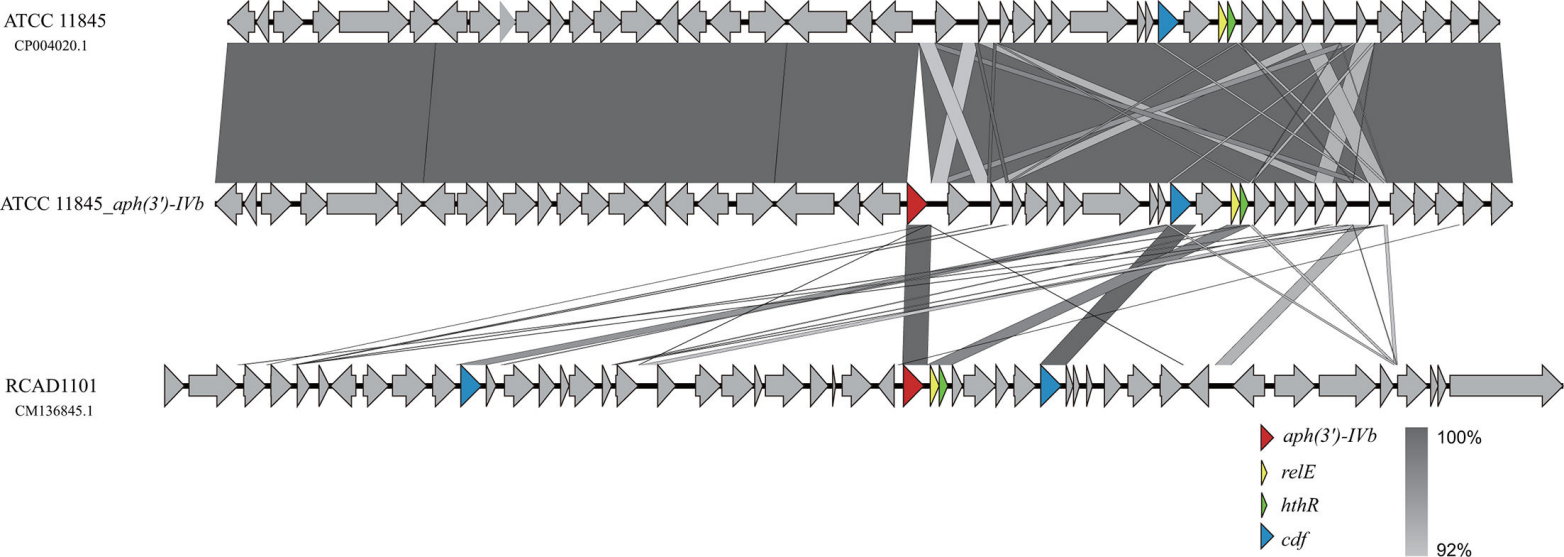
Natural transformation of *aph(3′)-IVb* into *R. anatipestifer* ATCC 11845 genome. The *aph(3′)-IVb* gene from RCAD1101 was inserted into the genome of ATCC 11845. Linear genomic comparison shows the genetic organization of the insertion site in the wild-type recipient ATCC 11845 (top), the constructed insertion mutant ATCC 11845_*aph(3′)-IVb* (middle), and the donor strain RCAD1101 (bottom). Arrows indicate coding sequences and their direction of transcription, color-coded by function: red arrows indicate the resistance gene *aph(3′)-IVb*; yellow arrows indicate the *relE* gene (encoding a type II toxin-antitoxin system toxin); green arrows indicate the *hthR* gene (encoding a putative HTH-type transcriptional regulator); and blue arrows indicate the *cdf* gene (encoding a cation diffusion facilitator family transporter). Gray-shaded regions denote sequence homology. The results confirm the successful site-specific integration of *aph(3′)-IVb* into the corresponding chromosomal locus of ATCC 11845 flanked by these conserved marker genes.

To investigate the potential for horizontal transfer of *aph(3*′*)-IVb* mediated by the natural competence of *R. anatipestifer*, we conducted natural transformation assays using both genomic DNA extracted from strain RCAD1101 and a PCR-amplified *aph(3*′*)-IVb* fragment. Neomycin-resistant transformants were successfully obtained using both donor materials. Sequence analysis revealed that the integration profiles were identical for transformants derived from both genomic DNA and the PCR fragment. Specifically, the 27 bp 5′ untranslated region (UTR) and 140 bp 3′ UTR of *aph(3*′*)-IVb* were consistently co-integrated along with the coding sequence (Fig. 3). Analysis of the 100 kb flanking region in ATCC 11845 using VRprofile2 (33)—covering prophages, GRIs, integrative and conjugative elements (ICEs), integrons, and transposons—revealed an absence of potential mobile genetic elements. Notably, this integration event occurred in the absence of flanking sequence homology with the donor fragment (Fig. 3), distinguishing it from canonical RecA-dependent homologous recombination (34, 35). This observation parallels findings reported for the *lnu*(H) (36) and *bla*_RATA_ (37) genes, implying that *R. anatipestifer* can integrate exogenous resistance determinants through a mechanism that has yet to be fully elucidated.

To further elucidate the potential mechanism underlying this homology-independent integration, we performed a multiple sequence alignment of the 140 bp downstream region across all analyzed *aph(3*′*)-IVb*-positive *R. anatipestifer* isolates, which revealed complete sequence identity. Secondary structure prediction of this segment using mfold (38) demonstrated the formation of stable stem-loop structures (Fig. S6). Notably, the folding topology of these structures exhibits structural features analogous to the canonical attC recombination sites recognized by integrons (39). This structural resemblance, coupled with the absence of flanking sequence homology, suggests that the acquisition of *aph(3*′*)-IVb* is likely mediated by a site-specific recombination mechanism relying on these conserved secondary structural motifs.

However, despite this demonstrated mobility, the prevalence of *aph(3*′*)-IVb* remains remarkably low (1.6%, 16/975) in the global *R. anatipestifer* population. This paradox may be explained by functional redundancy. Given that *R. anatipestifer* inherently possesses robust multidrug efflux systems, such as the RaeABCR and RaeE-RaeF-RopN complexes, and the RanARanB transporter (12, 14, 16), the species exhibits a high basal tolerance to aminoglycosides. Consequently, it may rely less on specific modifying enzymes for survival under standard selection pressures.

Finally, analysis of the broader distribution of *aph(3*′*)-IVb* revealed that it is detected across diverse pathogenic species, particularly those affecting humans. To investigate the genetic context in these pathogens, we analyzed the 10 kb sequences upstream and downstream of the gene (Fig. S5). The results showed that a plethora of resistance genes, as well as ICEs, were frequently co-localized with *aph(3*′*)-IVb*. This was especially notable in *Glaesserella parasuis* strain LSR011 (isolated from *Sus scrofa domesticus* with polyserositis), where the gene was embedded within a complex mobile region. We postulate that *aph(3*′*)-IVb* exploits the mobility of these elements via a mechanism of genetic hitchhiking. By integrating into ICEs, the gene leverages their intrinsic conjugative machinery to facilitate efficient interspecies dissemination. The association with ICEs suggests that *aph(3*′*)-IVb* possesses high transferability potential. The strong dissemination capacity of these elements likely contributes to the global distribution of the gene across the human-animal interface, underscoring the potential threat it poses to public health.

### Conclusion

The identification of *aph(3*′*)-IVb* represents a significant expansion of the APH(3′)-IV subclass, providing novel insights into the evolutionary plasticity of aminoglycoside-modifying enzymes. The preservation of the resistance phenotype, despite substantial sequence divergence (~40% identity to APH(3′)-IVa), suggests that the diversity of this enzyme family is far more extensive than previously recognized. Moreover, the demonstrated acquisition of *aph(3*′*)-IVb* via natural transformation exemplifies the remarkable genomic plasticity of *R. anatipestifer*, highlighting its capacity to actively capture and integrate exogenous determinants in response to antimicrobial pressure. Mechanistically, unlike determinants that confer high-level resistance in isolation, APH(3′)-IVb functions as a “resistance amplifier,” synergizing with intrinsic efflux systems to elevate basal tolerance to clinically significant resistance levels. Furthermore, the localization of *aph(3*′*)-IVb* within a mobile genomic resistance island, coupled with its detection in diverse human pathogens, reveals a critical pathway for resistance dissemination across the human-animal interface. These findings underscore the urgency of adopting a “One Health” strategy to surveil such cryptic resistance elements and mitigate their potential public health impact.

## MATERIALS AND METHODS

### Bacterial strains, plasmids, and culture conditions

Table S1 summarizes the bacterial strains, plasmids, and primers used in this study. *R. anatipestifer* strain RCAD1101 was isolated in September 2020 from the respiratory tract of a diseased duck in Yunnan Province, China. Clinical samples were streaked onto Tryptic Soy Agar (TSA; Oxoid Ltd., Basingstoke, UK) supplemented with 5% sheep blood and incubated at 37°C. Individual colonies were purified and identified via Sanger sequencing of the full-length ^16^S rRNA gene (40).

*E. coli* DH5α was utilized as the host for routine cloning, while *E. coli* BL21(DE3) served as the expression host for the purification of APH(3′)-IVb. The multidrug-susceptible strain *R. anatipestifer* ATCC 11845 was used as the recipient for natural transformation experiments. Plasmids pET32a and pLMF03 were employed as cloning and expression vectors. The pET28a vector was specifically used for the overexpression and purification of His-tagged APH(3′)-IVb. Unless otherwise specified, bacterial strains were routinely cultured overnight in Luria-Bertani broth at 37°C, supplemented with appropriate antimicrobial agents and, when required, solidified with 1.5% agar.

### Nucleotide and protein sequence analysis

Genomic DNA was extracted from *R. anatipestifer* RCAD1101 using the TIANamp Bacteria DNA Kit (Tiangen, China). Sequencing libraries were prepared using the MGIEasy DNA Library Prep Kit (MGI Tech Co., Ltd., Shenzhen, China) and the ONT 1D Ligation Sequencing Kit (SQK-LSK109). Sequencing was subsequently performed on the MGISEQ-2000 (BGI, Shenzhen, China) and Oxford Nanopore MinION (Oxford Nanopore Technologies, UK) platforms, respectively, in accordance with the manufacturers’ protocols. The hybrid assembly was generated using Canu (v1.5) with default parameters, followed by two rounds of polishing with Pilon (v1.24) utilizing the short-read data (41, 42).

Putative resistance determinants in RCAD1101 were identified using AMRFinderPlus (version 3.10.24) (17). Multiple sequence alignments were performed using CLUSTAL W (43) and visualized using ESPRipt 3.0 (44), and phylogenetic trees were constructed using MEGA11 (45). The three-dimensional structure of APH(3′)-IVb was predicted using AlphaFold 3 (46). Molecular docking studies with ATP and aminoglycoside substrates were conducted using AutoDock Vina (47). The genetic environment was visualized and analyzed using EasyFig (48).

### Cloning, expression, and purification of the APH(3′)-IVb protein

The *aph(3*′*)-IVb* gene was cloned into the pET32a and pLMF03 vectors using a seamless cloning strategy (Sangon Biotech, Shanghai, China). The resulting recombinant plasmids, pET32a-*aph(3*′*)-IVb* and pLMF03-*aph(3*′*)-IVb*, were verified by Sanger sequencing (Sangon Biotech) and subsequently transformed into *E. coli* BL21(DE3) and *R. anatipestifer* ATCC 11845, respectively.

For protein characterization, APH(3′)-IVb was overexpressed in *E. coli* BL21(DE3) using the pET28a expression system. Briefly, the *aph(3*′*)-IVb* coding sequence was inserted into pET28a to generate an N-terminal His_6_-tagged fusion protein containing a thrombin cleavage site. Cultures were grown at 37°C until the optical density at 600 nm (OD_600_) reached 0.6–0.8. Expression was induced by the addition of isopropyl β-D-1-thiogalactopyranoside to a final concentration of 0.5 mM, followed by incubation for 24 h at 25°C. Cells were harvested by centrifugation (5,000 × *g*, 10 min, 4°C), resuspended in lysis buffer (20 mM Tris-HCl, 150 mM NaCl, 3 mM β-mercaptoethanol, 0.5% NP-40, pH 8.0), and disrupted via sonication. Cellular debris was removed by centrifugation (10,000 × *g*, 30 min, 4°C). The supernatant was incubated with pre-equilibrated Ni-NTA agarose (Beyotime, Shanghai, China) for 8 h at 4°C with gentle agitation, followed by purification via affinity chromatography. The His_6_ tag was excised by thrombin digestion (25°C, 3 h), and the reaction mixture was passed through a Ni-NTA column to remove free tags and uncleaved protein. Protein purity was assessed via SDS-PAGE, and concentrations were determined spectrophotometrically and using the BCA Protein Assay Kit (Thermo Fisher Scientific, Waltham, MA, USA).

### Construction of deletion strain RCAD1101Δ*aph(3*′*)-IVb*, complementation strain RCAD1101Δ*aph(3*′*)-IVb*(pLMF03-*aph(3*′*)-IVb*), and site-directed mutagenesis

The deletion strain RCAD1101Δ*aph(3*′*)-IVb* was generated using an unmarked gene deletion strategy (49), with minor modifications. Briefly, the upstream (*aph(3*′*)-IVb*L, 800 bp) and downstream (*aph(3*′*)-IVb*R, 800 bp) fragments of the *aph(3*′*)-IVb* gene were amplified from RCAD1101 genomic DNA using the primers *aph(3′)-IVb*L-F1/*aph(3*′*)-IVb*L-R1 and *aph(3*′*)-IVb*R-F1/*aph(3*′*)-IVb*R-R1 (Table S1), respectively. Concurrently, the *cfx*A-SacB cassette was amplified from the plasmid pBAD24::*cfx*A-SacB using the primers *cfx*A-SacB-F/*cfx*A-SacB-R. These three fragments were assembled using a seamless cloning kit to generate the fusion fragment *aph(3*′*)- IVb*L-*cfx*A-SacB-*aph(3*′*)- IVb*R fragment which was subsequently amplified using primers *aph(3*′*)- IVb*L-F1 and *aph(3*′*)- IVb*R-R2.

The purified fusion fragment (2 μg) was incubated with 1 mL of RCAD1101 suspension (OD_600_ = 1) for 12 h to induce natural transformation, as described previously (34). Transformants were selected on TSA plates containing cefoxitin (1 mg/L), and the intermediate mutant strain RCAD1101Δ*aph(3*′*)-IVb*::cfx-sacB was isolated. Subsequently, the 1,000 bp upstream fragment (up) and 1,000 bp downstream (down) fragments of *aph(3*′*)-IVb* were amplified with the primers *aph(3*′*)-IVb*L-F2/*aph(3′)-IVb*-overlap and *aph(3*′*)-IVb*R-F/ *aph(3*′*)-IVb*-R2. These two PCR fragments were assembled using the seamless cloning kit. The resulting up-down fragment was incubated with the intermediate mutant RCAD1101Δ*aph(3*′*)-IVb*::*cfx*A-sacB for 12 h and counter-selected on a TSA plate containing 15% sucrose to obtain the final deletion mutant RCAD1101Δ*aph(3*′*)-IVb*.

To construct the complementation strain, the recombinant plasmid pLMF03-*aph(3*′*)-IVb* was introduced into the mutant strain RCAD1101Δ*aph(3*′*)-IVb* via natural transformation as described above. Transformants were selected on TSA plates supplemented with cefoxitin (1 mg/L) and confirmed by PCR analysis. The resulting complementation strain was designated RCAD1101*Δaph(3*′*)-IVb*(*pLMF03-aph(3*′*)-IVb*).

Site-directed mutants were further constructed using *in vitro* site-directed mutagenesis (26). The upstream and downstream fragments of the target *aph(3*′*)-IVb* gene were amplified using mutagenic primers (Table S1). The amplified fragments were fused by overlap extension PCR to generate mutant alleles, which were subsequently cloned into the pET32a vector for expression and purification as described in “Cloning, expression, and purification of the APH(3′)-IVb protein.”

### Antimicrobial susceptibility testing

Minimum inhibitory concentrations (MICs) were determined using the standard broth microdilution method in accordance with Clinical and Laboratory Standards Institute guidelines (50). Notably, butirosin was excluded from the testing panel due to its commercial unavailability. *E. coli* ATCC 25922 served as the quality-control strain.

Given the absence of standardized antibiotic breakpoints for *R. anatipestifer*, strain ATCC 11845 was utilized as the reference standard to assess the resistance phenotype of RCAD1101. This selection was based on the fact that ATCC 11845 harbors no known resistance determinants other than the intrinsic aminoglycoside efflux pumps (12, 14). The inoculum density was adjusted to approximately 10^7^ CFU/mL (100 μL per well). Wells containing inoculated broth without antibiotics served as growth controls, while uninoculated wells served as sterility controls. All assays were performed in triplicate.

### Enzyme kinetics

The enzyme kinetics of APH(3′)-IVb were assessed using a previously described assay (51, 52) with minor modifications. Briefly, kinetic parameters were determined using a continuous spectrophotometric assay that couples ADP production from aminoglycoside phosphorylation to NADH oxidation via pyruvate kinase (PK) and lactate dehydrogenase (LD). ADP production was quantified by monitoring the decrease in absorbance at 340 nm with a UV-VIS spectrophotometer (U-3900, Hitachi, Japan) at 25°C.

Reactions were initiated by adding APH(3′)- IVb (final concentration 150 nM) to a 250 μL mixture containing 100 mM HEPES (pH 7.0), 10 mM MgCl_2_, 20 mM KCl, 2 mM phosphoenolpyruvate, 100 μM NADH, a commercial PK/LD enzyme mixture (Sigma P0294; 18–26 U/mL PK and 25–35 U/mL LD), 2 mM ATP, and aminoglycosides at various concentrations. Steady-state velocities were calculated from the linear phase of reaction progress curves and plotted against substrate concentration. Data were fitted to the Michaelis-Menten equation, *v* = (*V*_max_[*S*])/( *K_m_* + [*S*]), by non-linear least-squares regression, using GraphPad Prism 9 (GraphPad Software, Inc., San Diego, CA, USA) to determine *K_m_* and *k*_cat_ values. In this equation, *v* represents the steady-state velocity, *V*_max_ the maximal velocity, [*S*] the substrate concentration, *K_m_* the Michaelis constant, and *k*_cat_ the turnover number, calculated from *V*_max_ = *k*_cat_ [*E*], where [*E*] is the enzyme concentration.

### Prevalence of *aph(3*′*)-IVb* in *R. anatipestifer* field isolates and its distribution across ecological niches

To investigate the prevalence of the *aph(3*′*)-IVb* gene in field isolates, 741 *R. anatipestifer* isolates maintained in our laboratory collection were screened via PCR using primers *aph(3*′*)-IVb*-F1 and *aph(3*′*)-IVb*-R1 (Table S1). PCR amplification was performed using 2× Taq Master Mix (Dye Plus; Vazyme, Nanjing, China) under the following conditions: initial denaturation at 95°C for 5 min, followed by 30 cycles of (95°C/30 s, 67°C/30 s, 72°C/45 s), and a final extension at 72°C for 8 min. All PCR products (approx. 789 bp) were verified by Sanger sequencing.

To characterize the global distribution of *aph(3*′*)-IVb*, we performed a BLASTp search of APH(3*′*)-IVb sequences against the NCBI Non-Redundant database (https://blast.ncbi.nlm.nih.gov/Blast.cgi), retrieving complete protein sequence hits with an identity threshold of >90%. Additionally, we screened publicly available genome sequences (*n* = 234, data as of 29 August 2025) from the NCBI RefSeq database (https://www.ncbi.nlm.nih.gov/refseq/) in conjunction with the *aph(3*′*)-IVb*-positive isolates identified in this study to assess the overall prevalence of *aph(3*′*)-IVb* within the *R. anatipestifer* population.

### Natural transformation of *aph(3*′*)-IVb* into *R. anatipestifer* ATCC 11845 genome

To investigate the transferability of the *aph(3*′*)-IVb* gene, we utilized the reference strain ATCC 11845 as the recipient host. The genomic DNA of *R. anatipestifer* RCAD1101 and the *aph(3*′*)-IVb* gene, along with its flanking 5′ and 3′ untranslated regions (UTRs), were prepared as donor materials (Table S1). Briefly, different concentrations of genomic DNA (0.5, 1, 5, 10, and 20 μg) or 2 μg of the purified PCR fragment were individually incubated with 1 mL of an ATCC 11845 suspension (OD_600_ = 1) for 12 h to induce natural transformation. Transformants were selected on Tryptic Soy Agar (TSA) plates supplemented with neomycin (256 mg/L), and the resulting strains were subsequently confirmed.

## Data Availability

The genome sequence of *R. anatipestifer* strain RCAD1101 was deposited in GenBank under accession number CM136845.1, while its 16S rRNA gene sequence has been assigned accession number PX365741.1. Additionally, the nucleotide sequence of the novel resistance gene *aph(3′)-IVb* was deposited under accession number PX763553.1.

## SUPPLEMENTAL MATERIAL

Supplemental figures aac.01631-25-s0001.docx Fig. S1 to S6.

Supplemental tables aac.01631-25-s0002.xlsx Tables S1 to S5.

ASM does not own the copyrights to Supplemental Material that may be linked to, or accessed through, an article. The authors have granted ASM a non-exclusive, world-wide license to publish the Supplemental Material files. Please contact the corresponding author directly for reuse.

## References

[1] Serio AW, Keepers T, Andrews L, Krause KM. 2018. Aminoglycoside revival: review of a historically important class of antimicrobials undergoing rejuvenation. EcoSal Plus 8. doi:10.1128/ecosalplus.ESP-0002-2018 PMC11575671 30447062

[2] Krause KM, Serio AW, Kane TR, Connolly LE. 2016. Aminoglycosides: an overview. Cold Spring Harb Perspect Med 6:a027029. doi:10.1101/cshperspect.a027029 27252397 PMC4888811

[3] Labby KJ, Garneau-Tsodikova S. 2013. Strategies to overcome the action of aminoglycoside-modifying enzymes for treating resistant bacterial infections. Future Med Chem 5:1285–1309. doi:10.4155/fmc.13.80 23859208 PMC3819198

[4] Garneau-Tsodikova S, Labby KJ. 2016. Mechanisms of resistance to aminoglycoside antibiotics: overview and perspectives. Medchemcomm 7:11–27. doi:10.1039/C5MD00344J 26877861 PMC4752126

[5] Ramirez MS, Tolmasky ME. 2010. Aminoglycoside modifying enzymes. Drug Resist Updat 13:151–171. doi:10.1016/j.drup.2010.08.003 20833577 PMC2992599

[6] Shaw KJ, Rather PN, Hare RS, Miller GH. 1993. Molecular genetics of aminoglycoside resistance genes and familial relationships of the aminoglycoside-modifying enzymes. Microbiol Rev 57:138–163. doi:10.1128/mr.57.1.138-163.1993 8385262 PMC372903

[7] Perlin MH, Brown SA, Dholakia JN. 1999. Developing a snapshot of the ATP binding domain(s) of aminoglycoside phosphotransferases. Front Biosci 4:D63–D71. doi:10.2741/perlin 9872732

[8] Wright GD, Thompson PR. 1999. Aminoglycoside phosphotransferases: proteins, structure, and mechanism. Front Biosci 4:D9–D21. doi:10.2741/wright 9872733

[9] Herbert CJ, Sarwar M, Ner SS, Giles IG, Akhtar M. 1986. Sequence and interspecies transfer of an aminoglycoside phosphotransferase gene (APH) of Bacillus circulans. Self-defence mechanism in antibiotic-producing organisms. Biochem J 233:383–393. doi:10.1042/bj2330383 3006668 PMC1153039

[10] García-López M, Meier-Kolthoff JP, Tindall BJ, Gronow S, Woyke T, Kyrpides NC, Hahnke RL, Göker M. 2019. Analysis of 1,000 type-strain genomes improves taxonomic classification of Bacteroidetes. Front Microbiol 10:2083. doi:10.3389/fmicb.2019.02083 31608019 PMC6767994

[11] Christensen JP, Bisgaard M. 2019. Pasteurellosis and other respiratory bacterial infections. In Swayne DE (ed), Diseases of poultry, 14th ed. John Wiley & Sons, Inc, Hoboken, NJ.

[12] Li S, Chen Q, Gong X, Liu Y, Zheng F. 2020. RanB, a putative ABC-type multidrug efflux transporter contributes to aminoglycosides resistance and organic solvents tolerance in Riemerella anatipestifer. Vet Microbiol 243:108641. doi:10.1016/j.vetmic.2020.108641 32273020

[13] Hao J, Zhang J, He X, Wang Y, Su J, Long J, Zhang L, Guo Z, Zheng Y, Wang M, Sun Y. 2025. Unveiling the silent threat: a comprehensive review of Riemerella anatipestifer – from pathogenesis to drug resistance. Poult Sci 104:104915. doi:10.1016/j.psj.2025.104915 40020410 PMC11919424

[14] Zhang X, Wang M-S, Liu M-F, Zhu D-K, Biville F, Jia R-Y, Chen S, Sun K-F, Yang Q, Wu Y, Zhao X-X, Chen X-Y, Cheng A-C. 2017. Contribution of RaeB, a putative RND-type transporter to aminoglycoside and detergent resistance in Riemerella anatipestifer. Front Microbiol 8:2435. doi:10.3389/fmicb.2017.02435 29276505 PMC5727081

[15] Murphy E. 1985. Nucleotide sequence of a spectinomycin adenyltransferase AAD(9) determinant from Staphylococcus aureus and its relationship to AAD(3") (9). Mol Gen Genet 200:33–39. doi:10.1007/BF00383309 2993813

[16] Wang Y, Li S, Gong X, Chen Q, Ji G, Liu Y, Zheng F. 2020. Characterization of RaeE-RaeF-RopN, a putative RND efflux pump system in Riemerella anatipestifer. Vet Microbiol 251:108852. doi:10.1016/j.vetmic.2020.108852 33069037

[17] Feldgarden M, Brover V, Gonzalez-Escalona N, Frye JG, Haendiges J, Haft DH, Hoffmann M, Pettengill JB, Prasad AB, Tillman GE, Tyson GH, Klimke W. 2021. AMRFinderPlus and the reference gene catalog facilitate examination of the genomic links among antimicrobial resistance, stress response, and virulence. Sci Rep 11:12728. doi:10.1038/s41598-021-91456-0 34135355 PMC8208984

[18] Boehr DD, Thompson PR, Wright GD. 2001. Molecular mechanism of aminoglycoside antibiotic kinase APH(3′)-IIIa. J Biol Chem 276:23929–23936. doi:10.1074/jbc.M100540200 11279088

[19] Kaul M, Barbieri CM, Srinivasan AR, Pilch DS. 2007. Molecular determinants of antibiotic recognition and resistance by aminoglycoside phosphotransferase (3′)-IIIa: a calorimetric and mutational analysis. J Mol Biol 369:142–156. doi:10.1016/j.jmb.2007.02.103 17418235 PMC2040079

[20] Nurizzo D, Shewry SC, Perlin MH, Brown SA, Dholakia JN, Fuchs RL, Deva T, Baker EN, Smith CA. 2003. The crystal structure of aminoglycoside-3’-phosphotransferase-IIa, an enzyme responsible for antibiotic resistance. J Mol Biol 327:491–506. doi:10.1016/s0022-2836(03)00121-9 12628253

[21] Chothia C, Lesk AM. 1986. The relation between the divergence of sequence and structure in proteins. EMBO J 5:823–826. doi:10.1002/j.1460-2075.1986.tb04288.x 3709526 PMC1166865

[22] Worth CL, Gong S, Blundell TL. 2009. Structural and functional constraints in the evolution of protein families. Nat Rev Mol Cell Biol 10:709–720. doi:10.1038/nrm2762 19756040

[23] Shi K, Caldwell SJ, Fong DH, Berghuis AM. 2013. Prospects for circumventing aminoglycoside kinase mediated antibiotic resistance. Front Cell Infect Microbiol 3:22. doi:10.3389/fcimb.2013.00022 23805415 PMC3691515

[24] Herbert CJ, Giles IG, Akhtar M. 1983. The sequence of an antibiotic resistance gene from an antibiotic-producing bacterium. Homologies with transposon genes. FEBS Lett 160:67–71. doi:10.1016/0014-5793(83)80937-5 6193008

[25] Lin N, Sha Y, Zhang G, Song C, Zhang Y, Zhao J, Huang D, Lu J, Bao Q, Pan W. 2024. APH(3’)-Ie, an aminoglycoside-modifying enzyme discovered in a rabbit-derived Citrobacter gillenii isolate. Front Cell Infect Microbiol 14:1435123. doi:10.3389/fcimb.2024.1435123 39139766 PMC11320999

[26] Huang L, Yuan H, Liu M-F, Zhao X-X, Wang M-S, Jia R-Y, Chen S, Sun K-F, Yang Q, Wu Y, Chen X-Y, Cheng A-C, Zhu D-K. 2017. Type B chloramphenicol acetyltransferases are responsible for chloramphenicol resistance in Riemerella anatipestifer, China. Front Microbiol 8:297. doi:10.3389/fmicb.2017.00297 28298905 PMC5331189

[27] Hon W-C, McKay GA, Thompson PR, Sweet RM, Yang DSC, Wright GD, Berghuis AM. 1997. Structure of an enzyme required for aminoglycoside antibiotic resistance reveals homology to eukaryotic protein kinases. Cell 89:887–895. doi:10.1016/s0092-8674(00)80274-3 9200607

[28] Takahashi H, Fujii T, Yamakawa S, Yamada C, Fujiki K, Kondo N, Funasaka K, Hirooka Y, Tochio T. 2023. Combined oral intake of short and long fructans alters the gut microbiota in food allergy model mice and contributes to food allergy prevention. BMC Microbiol 23:266. doi:10.1186/s12866-023-03021-6 37737162 PMC10515425

[29] Zhang R, Dong N, Shen Z, Zeng Y, Lu J, Liu C, Zhou H, Hu Y, Sun Q, Cheng Q, Shu L, Cai J, Chan EW-C, Chen G, Chen S. 2020. Epidemiological and phylogenetic analysis reveals Flavobacteriaceae as potential ancestral source of tigecycline resistance gene tet(X). Nat Commun 11:4648. doi:10.1038/s41467-020-18475-9 32938927 PMC7494873

[30] Cheng Y-Y, Liu Y, Chen Y, Huang F-M, Chen R-C, Xiao Y-H, Zhou K. 2021. Sporadic dissemination of tet(X3) and tet(X6) mediated by highly diverse plasmidomes among livestock-associated Acinetobacter. Microbiol Spectr 9:e0114121. doi:10.1128/Spectrum.01141-21 34851156 PMC8635130

[31] Dong N, Zeng Y, Cai C, Sun C, Lu J, Liu C, Zhou H, Sun Q, Shu L, Wang H, Wang Y, Wang S, Wu C, Chan EW-C, Chen G, Shen Z, Chen S, Zhang R. 2022. Prevalence, transmission, and molecular epidemiology of tet(X)-positive bacteria among humans, animals, and environmental niches in China: an epidemiological, and genomic-based study. Sci Total Environ 818:151767. doi:10.1016/j.scitotenv.2021.151767 34801490

[32] Yan Z, Wang P, Wang H, Zhang J, Zhang Y, Wu Y, Zhou H, Li Y, Shen Z, Chen G, Li R, Zhang R. 2024. Emergence and genomic epidemiology of tigecycline resistant bacteria of fly origin across urban and rural China. Environ Int 193:109099. doi:10.1016/j.envint.2024.109099 39476596

[33] Wang M, Goh Y-X, Tai C, Wang H, Deng Z, Ou H-Y. 2022. VRprofile2: detection of antibiotic resistance-associated mobilome in bacterial pathogens. Nucleic Acids Res 50:W768–W773. doi:10.1093/nar/gkac321 35524563 PMC9252795

[34] Liu M, Zhang L, Huang L, Biville F, Zhu D, Wang M, Jia R, Chen S, Sun K, Yang Q, Wu Y, Chen X, Cheng A. 2017. Use of natural transformation to establish an easy knockout method in Riemerella anatipestifer. Appl Environ Microbiol 83:e00127-17. doi:10.1128/AEM.00127-17 28258143 PMC5394337

[35] Huang L, Tian X, Liu M, Wang M, Biville F, Cheng A, Zhu D, Jia R, Chen S, Zhao X, Yang Q, Wu Y, Zhang S, Huang J, Tian B, Yu Y, Liu Y, Zhang L, Pan L, Rehman MU, Chen X. 2019. DprA is essential for natural competence in Riemerella anatipestifer and has a conserved evolutionary mechanism. Front Genet 10:429. doi:10.3389/fgene.2019.00429 31156696 PMC6533540

[36] Luo H-Y, Liu M-F, Wang M-S, Zhao X-X, Jia R-Y, Chen S, Sun K-F, Yang Q, Wu Y, Chen X-Y, Biville F, Zou Y-F, Jing B, Cheng A-C, Zhu D-K. 2018. A novel resistance gene, lnu(H), conferring resistance to lincosamides in Riemerella anatipestifer CH-2. Int J Antimicrob Agents 51:136–139. doi:10.1016/j.ijantimicag.2017.08.022 28843817

[37] Luo H, Yang Z, Lei T, Li C, Zhou Z, Wang M, Zhu D, Li P, Cheng A. 2024. RATA: a novel class a carbapenemase with broad geographic distribution and potential for global spread. Sci Total Environ 931:172873. doi:10.1016/j.scitotenv.2024.172873 38692330

[38] Zuker M. 2003. Mfold web server for nucleic acid folding and hybridization prediction. Nucleic Acids Res 31:3406–3415. doi:10.1093/nar/gkg595 12824337 PMC169194

[39] Nivina A, Escudero JA, Vit C, Mazel D, Loot C. 2016. Efficiency of integron cassette insertion in correct orientation is ensured by the interplay of the three unpaired features of attC recombination sites. Nucleic Acids Res 44:7792–7803. doi:10.1093/nar/gkw646 27496283 PMC5027507

[40] Zhu D-K, Yang X-Q, He Y, Zhou W-S, Song X-H, Wang J-B, Zhang Y, Liu M-F, Wang M-S, Jia R-Y, Chen S, Sun K-F, Yang Q, Wu Y, Chen X-Y, Cheng A-C. 2016. Comparative genomic analysis identifies structural features of CRISPR-Cas systems in Riemerella anatipestifer. BMC Genomics 17:689. doi:10.1186/s12864-016-3040-4 27577199 PMC5006608

[41] Walker BJ, Abeel T, Shea T, Priest M, Abouelliel A, Sakthikumar S, Cuomo CA, Zeng Q, Wortman J, Young SK, Earl AM. 2014. Pilon: an integrated tool for comprehensive microbial variant detection and genome assembly improvement. PLoS One 9:e112963. doi:10.1371/journal.pone.0112963 25409509 PMC4237348

[42] Koren S, Walenz BP, Berlin K, Miller JR, Bergman NH, Phillippy AM. 2017. Canu: scalable and accurate long-read assembly via adaptive k-mer weighting and repeat separation. Genome Res 27:722–736. doi:10.1101/gr.215087.116 28298431 PMC5411767

[43] Thompson JD, Higgins DG, Gibson TJ. 1994. CLUSTAL W: improving the sensitivity of progressive multiple sequence alignment through sequence weighting, position-specific gap penalties and weight matrix choice. Nucleic Acids Res 22:4673–4680. doi:10.1093/nar/22.22.4673 7984417 PMC308517

[44] Robert X, Gouet P. 2014. Deciphering key features in protein structures with the new ENDscript server. Nucleic Acids Res 42:W320–W324. doi:10.1093/nar/gku316 24753421 PMC4086106

[45] Tamura K, Stecher G, Kumar S. 2021. MEGA11: molecular evolutionary genetics analysis version 11. Mol Biol Evol 38:3022–3027. doi:10.1093/molbev/msab120 33892491 PMC8233496

[46] Abramson J, Adler J, Dunger J, Evans R, Green T, Pritzel A, Ronneberger O, Willmore L, Ballard AJ, Bambrick J, et al.. 2024. Accurate structure prediction of biomolecular interactions with AlphaFold 3. Nature 630:493–500. doi:10.1038/s41586-024-07487-w 38718835 PMC11168924

[47] Trott O, Olson AJ. 2010. AutoDock Vina: improving the speed and accuracy of docking with a new scoring function, efficient optimization, and multithreading. J Comput Chem 31:455–461. doi:10.1002/jcc.21334 19499576 PMC3041641

[48] Sullivan MJ, Petty NK, Beatson SA. 2011. Easyfig: a genome comparison visualizer. Bioinformatics 27:1009–1010. doi:10.1093/bioinformatics/btr039 21278367 PMC3065679

[49] Tian X, Huang L, Wang M, Biville F, Zhu D, Jia R, Chen S, Zhao X, Yang Q, Wu Y, Zhang S, Huang J, Zhang L, Yu Y, Cheng A, Liu M. 2020. The functional identification of Dps in oxidative stress resistance and virulence of Riemerella anatipestifer CH-1 using a new unmarked gene deletion strategy. Vet Microbiol 247:108730. doi:10.1016/j.vetmic.2020.108730 32768200

[50] Weinstein MP, Lewis JS. 2020. The clinical and laboratory standards institute subcommittee on antimicrobial susceptibility testing: background, organization, functions, and processes. J Clin Microbiol 58:e01864-19. doi:10.1128/JCM.01864-19 31915289 PMC7041576

[51] Shi W, Lu J, Feng C, Gao M, Li A, Liu S, Zhang L, Zhang X, Li Q, Lin H, Lin X, Li K, Zhang H, Hu Y, Wang G, Bao Q, Jiang W. 2022. Functional characterization of a novel aminoglycoside phosphotransferase, APH(9)-Ic, and its variant from Stenotrophomonas maltophilia. Front Cell Infect Microbiol 12:1097561. doi:10.3389/fcimb.2022.1097561 36699730 PMC9868417

[52] Lu W, Li K, Huang J, Sun Z, Li A, Liu H, Zhou D, Lin H, Zhang X, Li Q, Lu J, Lin X, Li P, Zhang H, Xu T, Bao Q. 2021. Identification and characteristics of a novel aminoglycoside phosphotransferase, APH(3′)-IId, from an MDR clinical isolate of Brucella intermedia. J Antimicrob Chemother 76:2787–2794. doi:10.1093/jac/dkab272 34329431

