# Supplementary material for: Identification and characteristics of a novel acquired aminoglycoside phosphotransferase, APH(3′)-IVb, from Riemerella anatipestifer
Source: Antimicrob Agents Chemother. 2026 Mar 24;70(5):e01631-25. doi: 10.1128/aac.01631-25 (PMC13148043; doi:10.1128/aac.01631-25)
Supplement: Supplemental figures — Fig. S1 to S6. [file aac.01631-25-s0001.docx]

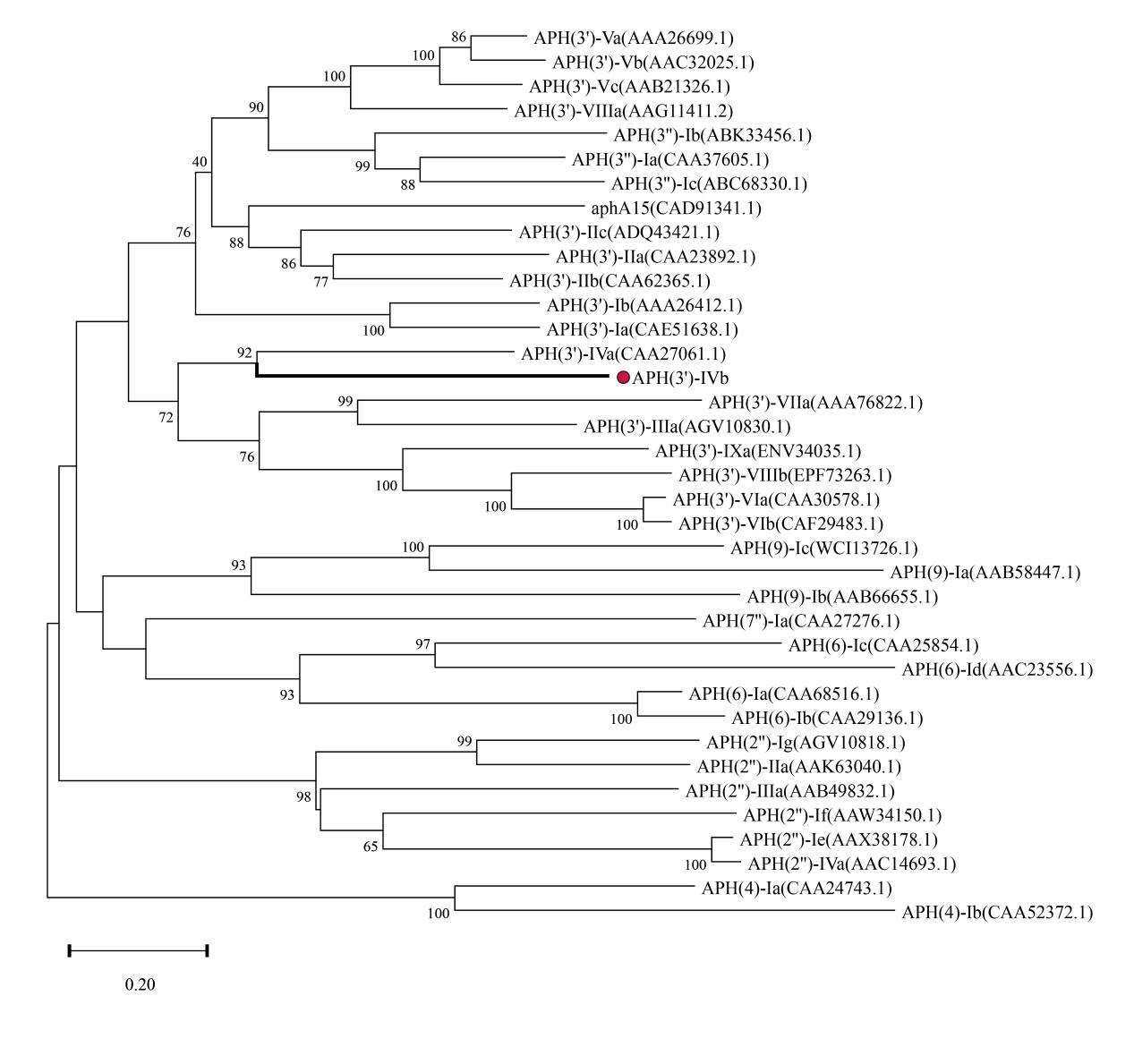


Figure S1. Phylogenetic analysis of APH(3')-IVb and other functionally characterized APH enzymes.

A phylogenetic tree showing the phylogenetic relationship between APH(3')-Ⅳb and other functionally characterized APHs. Among them, the APH(3')-Ⅳb is highlighted with red circle and bold line.


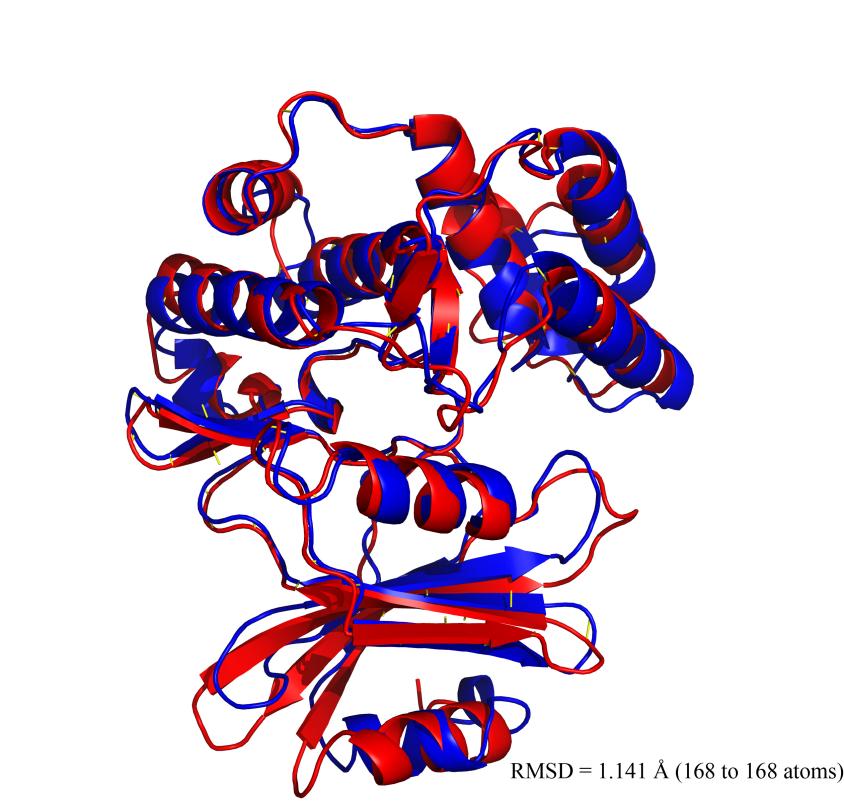


Figure S2. Structural superposition of the APH(3')-IVb and APH(3')-IVa proteins.

The three-dimensional structures of APH(3')-IVb (red, this study) and the reference enzyme APH(3')-IVa (blue) were predicted using AlphaFold 3. Structural alignment was performed using PyMOL. The superposition indicates a Root Mean Square Deviation (RMSD) of 1.141 Å calculated over 168 aligned atoms.


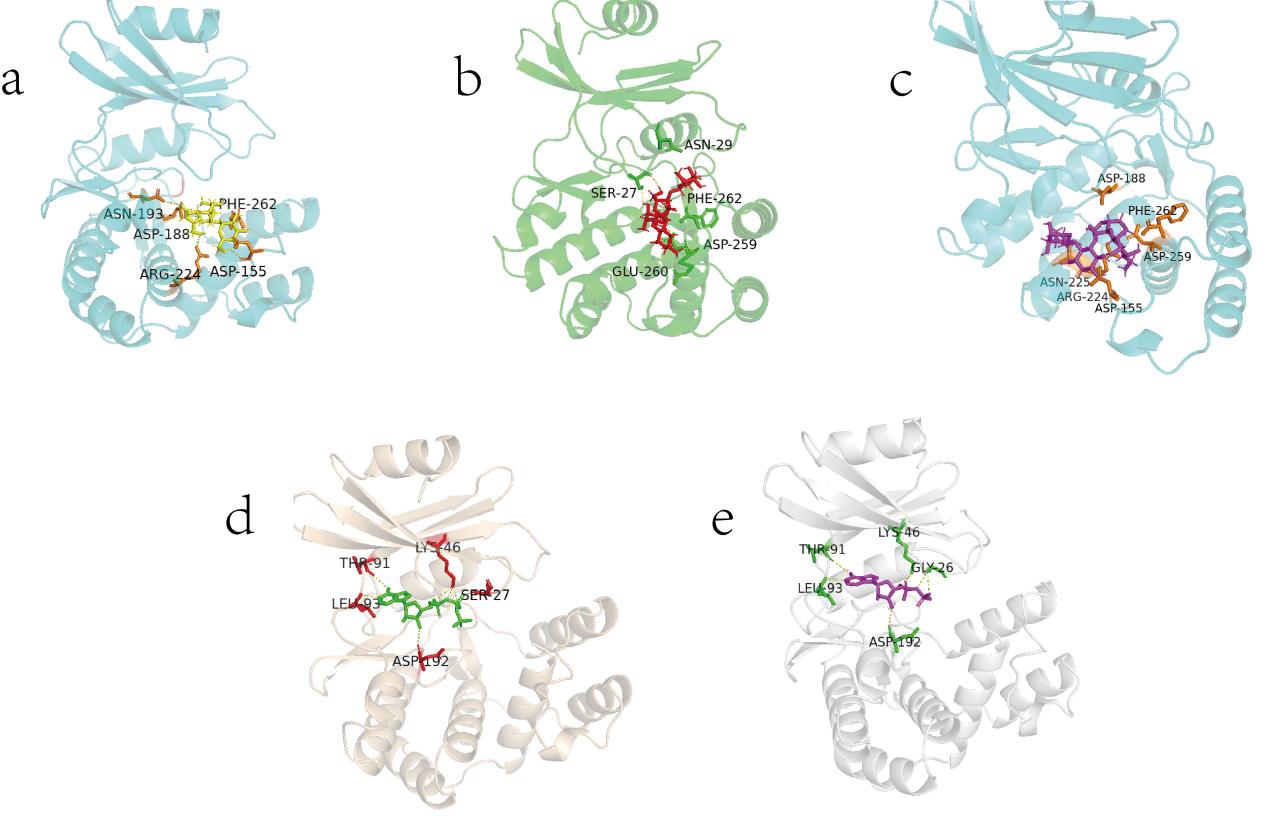


Figure S3. Molecular docking of APH(3')-Ⅳb

Binding schematic diagrams of the APH(3')-Ⅳb model with ribostamycin, paromomycin, neomycin, ATP, and ADP. (a) Binding schematic diagram of the APH(3')-Ⅳb model with ribostamycin; (b) Binding schematic diagram of the APH(3')-Ⅳb model with paromomycin; (c) Binding schematic diagram of the APH(3')-Ⅳb model with neomycin; (d) Binding schematic diagram of the APH(3')-Ⅳb model with ATP; (e) Binding schematic diagram of the APH(3')-Ⅳb model with ADP.


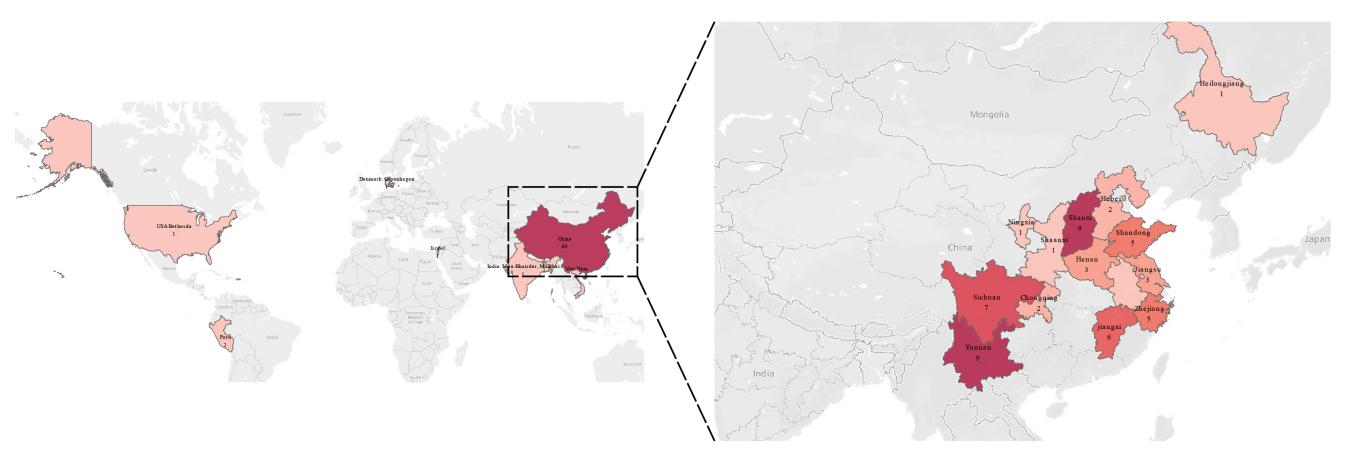


Figure S4. Geographical distribution of aph(3')-IVb-positive strains from public databases.

The strains were derived from aph(3')-IVb-positive strains retrieved from public databases, as listed in Table S5.


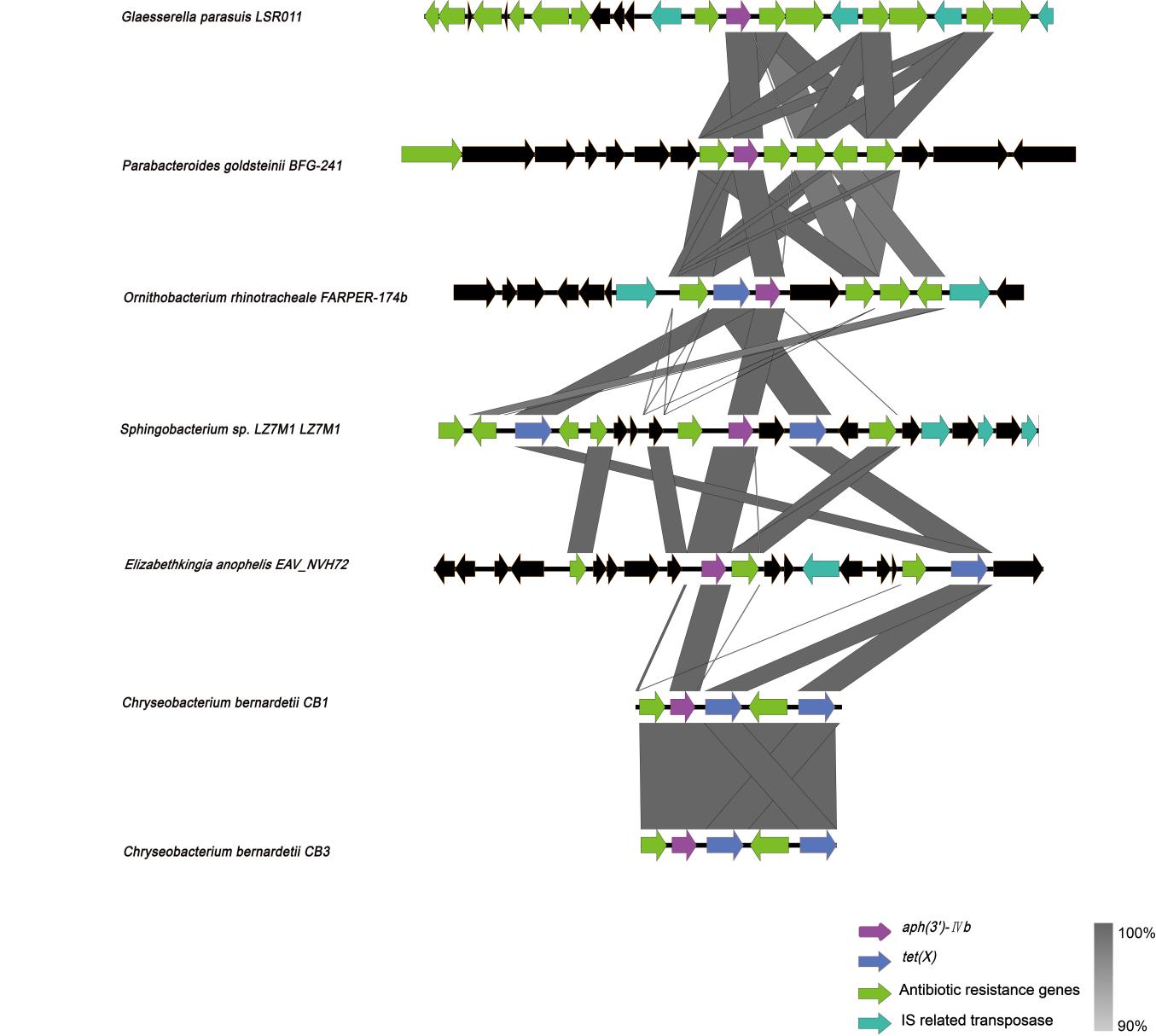


Figure S5. Genetic context of the aph(3′)-Ⅳb gene in aph(3′)-Ⅳb-positive strains from public databases.

Schematic representation of the genetic environment of aph(3′)-Ⅳb and comparison of the aph(3′)-Ⅳb-carrying regions in genomes of aph(3′)-Ⅳb-positive strains from public databases. Open reading frames (ORFs) are shown as arrows drawn to scale to indicate the direction of transcription. The aph(3′)-Ⅳb gene is coloured in purple, the tet(X) gene is coloured in dark blue, the known resistance genes are coloured in green, and the mobile elements are coloured in sky blue.


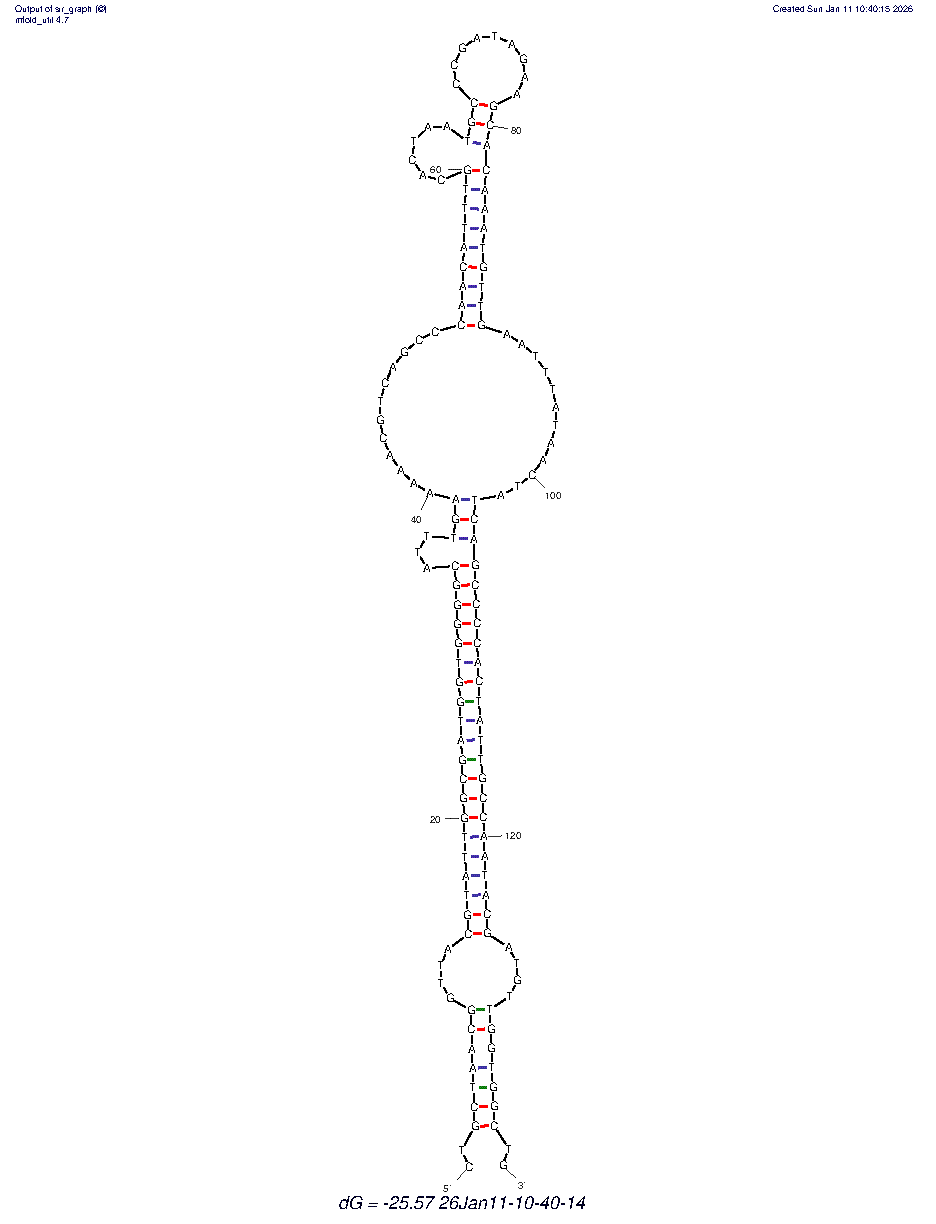


Figure S6. Predicted secondary structure of the conserved 140 bp downstream region of *aph(3')-IVb*.

The DNA secondary structure of the 140 bp sequence, which showed complete sequence identity across all analyzed *aph(3')-IVb*-positive *R. anatipestifer* isolates, was predicted using the mfold web server. The displayed structure represents the thermodynamically optimal folding configuration with the minimum free energy (ΔG = -25.57kcal/mol). The stable stem-loop structures exhibit structural features analogous to the canonical attC recombination sites recognized by integrons.
